# Vitamin D: A Bridge between Kidney and Heart

**DOI:** 10.3390/life14050617

**Published:** 2024-05-10

**Authors:** Carmine Secondulfo, Valeria Visco, Nicola Virtuoso, Martino Fortunato, Serena Migliarino, Antonella Rispoli, Lucia La Mura, Adolfo Stellato, Giuseppe Caliendo, Emanuela Settembre, Fabiana Galluccio, Sarah Hamzeh, Giancarlo Bilancio

**Affiliations:** 1 Department “Scuola Medica Salernitana”, University of Salerno, 84081 Baronissi, Italy; 2 Cardiology Unit, Salerno University Hospital “San Giovanni di Dio e Ruggi d’Aragona”, 84131 Salerno, Italy; 3 Centro Medico Ascione Srl, 80059 Torre del Greco, Italy; 4 Department of Medicine and Surgery, University of Naples “Federico II”, 80138 Napoli, Italy; 5 Nephrology Unit, Salerno University Hospital “San Giovanni di Dio e Ruggi d’Aragona”, 84131 Salerno, Italy

**Keywords:** vitamin D, calcitriol, calcidiol, kidney disease, cardiovascular disease, hypertension, osteoporosis, mineral bone disease, metabolic disease

## Abstract

Chronic kidney disease (CKD) and cardiovascular disease (CVD) are highly prevalent conditions, each significantly contributing to the global burden of morbidity and mortality. CVD and CKD share a great number of common risk factors, such as hypertension, diabetes, obesity, and smoking, among others. Their relationship extends beyond these factors, encompassing intricate interplay between the two systems. Within this complex network of pathophysiological processes, vitamin D has emerged as a potential linchpin, exerting influence over diverse physiological pathways implicated in both CKD and CVD. In recent years, scientific exploration has unveiled a close connection between these two prevalent conditions and vitamin D, a crucial hormone traditionally recognized for its role in bone health. This article aims to provide an extensive review of vitamin D’s multifaceted and expanding actions concerning its involvement in CKD and CVD.

## 1. Introduction

Chronic kidney disease (CKD) is a widespread health condition, commonly occurring and associated with a significant burden and significant morbidity. Globally, it has been documented with 697.5 million cases, representing a prevalence of 9.1%. CKD contributes to 35.8 million Disability-Adjusted Life Years (DALYs) and 1.2 million deaths in 2017 [1]. Although chronic kidney disease (CKD) is both preventable and treatable, its incidence is on the rise among the general population. Between 1990 and 2017, its prevalence surged by 29.3%, while the overall mortality rate increased by 41.5%. These statistics correspond with current forecasts, which predict CKD to become the fifth leading cause of global mortality by 2024 [2].

On the other hand, cardiovascular disease (CVD), despite a continuous expansion of biomedical knowledge and a constant effort in prevention and treatment, remains the primary cause of mortality and morbidity in Western countries [3].

CVD and CKD share many common risk factors, such as diabetes, hypertension, smoking, and obesity [4,5,6], and even some protective factors [7]. However, their relation is not limited to a number of common pre-existing predisposing conditions, but it is rooted in a more complex and interlinked mutual cross-talk [8].

Individuals afflicted with chronic kidney disease frequently face heightened susceptibility to cardiovascular complications, including coronary artery disease, heart failure, arrhythmias, and sudden cardiac death [9,10]. Although individuals in early stages of chronic kidney disease (CKD) (stages 1–3) already exhibit a higher occurrence and prevalence of cardiovascular events compared to the general population, those in advanced stages (stages 4–5) face an even greater risk. Among this high-risk group, cardiovascular complications, rather than end-stage kidney disease (CKD stage 5), emerge as the primary cause of mortality [11]. Traditional cardiovascular determinants present in CKD are not able to justify this excess risk in CKD, which appears to be an independent CVD risk factor itself [12]. Some evidence suggests that, among the main alterations caused by CKD, accelerated atherosclerotic degeneration and the development of vascular calcification are linked to worse prognosis [13,14]. This could possibly provide an explanatory mechanism for increased CVD damage in CKD patients [15,16].

In this context, vitamin D naturally emerges as a key factor in promoting both calcium/phosphorus metabolism imbalance, and thus CKD-related vascular calcification, and atherosclerosis, with a great impact on cardiovascular health [17,18]. In this review, the main roles of vitamin D in kidney and cardiovascular disease will be described.

## 2. What Is Vitamin D?

Vitamin D is a secosteroid, a steroid hormone obtained through dietary intake and by endogenous synthesis requiring exposure to sunlight. Essential vitamins are defined as substances that a living organism cannot produce adequately on its own and must acquire exclusively from its diet; for this reason, vitamin D it is not a true “vitamin”. There are six distinct steroid hormones referred to as vitamin D, each with different levels of activity. These include the endogenous precursor cholecalciferol (D3), derived from cholesterol; its partially active hydroxylated form, calcidiol (25(OH)D3), synthesized by the liver; and its active dihydroxy form, calcitriol (1,25(OH)2D3), hydroxylated in the kidneys [19,20]. Additionally, there is a plant-derived form known as ergocalciferol (D2), characterized by a worse pharmacokinetic profile, less biological activity, and lower stability than its animal-derived analogues [21,22]. Calcitriol’s most known effect is enhancing the absorption of calcium in the intestines and controlling phosphate levels. Vitamin D nuclear receptors (VDRs) are also present in a plethora of tissues, such as breast, brain, breast, lymphocyte and other immune cells, and prostate [23]; thus, it is unsurprising that vitamin D has various pleiotropic effects that are currently still under investigation, such as immune modulation, the onset of cancer, and insulin regulation [24,25]; many other cardiovascular regulatory functions will be described in greater detail in dedicated sections of this article.

**Figure 1:**
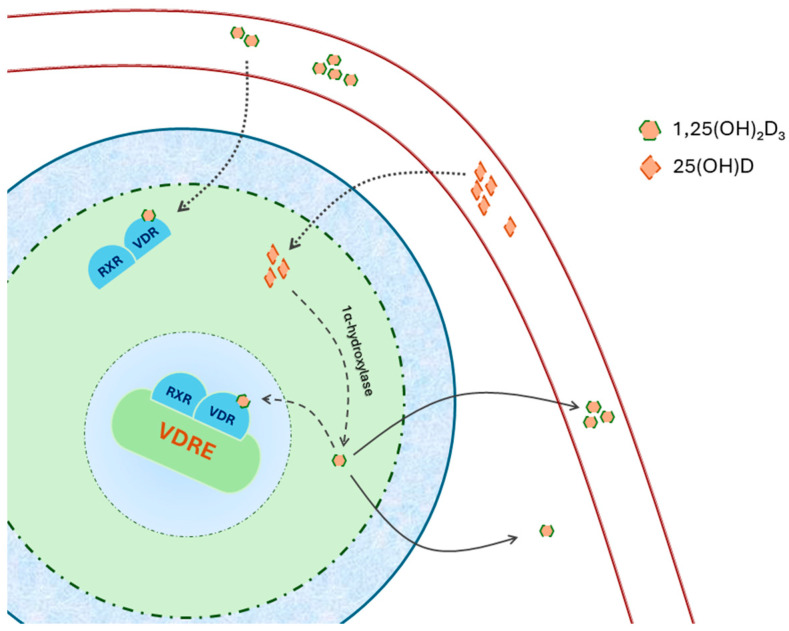
The dimer formed by 1,25(OH)2D3–VDR2 interacts with the retinoid X receptor (RXR) and translocates into the nucleus. Within the nucleus, it attaches to vitamin D response elements (VDRE) found in the promoter region of specific genes. 25(OH)D obtained from the bloodstream can be converted locally into 1,25(OH) D within cells expressing 1α-hydroxylase. Adapted from Latic [29].

The binding of calcitriol with VDR causes a conformational change in the receptor, leading to its heterodimerization with the retinoic acid X receptor (RXR) (Figure 1). Additionally, VDR can form heterodimers with other members of the steroid receptor gene family [26]. The transactivation of VDR results in the expression or repression of numerous genes, with estimates suggesting that calcitriol influences over 200 genes directly or indirectly, impacting a diverse array of physiological processes [27]. Notably, VDR–DNA binding aids in targeting genes that may undergo further modification by calcitriol. However, it is important to note that in many instances, changes in gene expression are not directly mediated by VDR but involve various co-regulatory elements [28]. These complexes typically include a VDR regulatory component and exhibit significant enzymatic activity [26].

### Vitamin D Deficiency

Vitamin D normal levels are not unanimously established, although many authors recognize that calcidiol serum levels <30 ng/mL can be described as “Vitamin D deficiency” [30,31]; levels below 10–12 ng/mL, associated with rickets and ostemalacia, are considered severe deficiency [32,33]. Furthermore, the clinical guidelines established by the Endocrine Society Task Force on Vitamin D have set a deficiency threshold for vitamin D at 50 nmol/L [30]. It must be noted that, while calcitriol is acknowledged as the most metabolically active vitamin D, its serum concentration is not regularly monitored. This is due to its short half-life, susceptibility to exogenous administration, and, most importantly, absence of a standardized assay. Consequently, calcidiol is the predominant biomarker utilized in both clinical and research settings [34]; however, calcidiol and calcitriol deficiency could impact mineral metabolism in different ways [35].

Recent data suggest that low vitamin D levels are common worldwide, varying across different ages and ethnicities, with a prevalence of 24% in the US and 40% in Europe, and over 20% in India and Pakistan [36,37]. In some groups of individuals, it can be even more common, such as in subjects with celiac disease or in obese and sedentary subjects [38,39]; in CKD patients, vitamin D deficiency prevalence can rise up to 85–99% [40,41].

There are some limitations that must be taken into account when pondering this information, hence the great disagreement in a generally acceptable definition of “normal values” of vitamin D and its deficiency [42]:

- No consensus on a standardized laboratory assay [43,44,45];
- Great variability in vitamin D levels among different populations and ethnicities, both due to genetic and geographical factors [46,47,48,49];
- Not clear whether the total or the free (unbound to carrier proteins) vitamin D should be measured [50,51].

## 3. Vitamin D in the Context of CKD

Chronic kidney disease is one of the main causes of vitamin D deficiency, and the progressive decline of renal function is associated with its worsening [52,53].

Vitamin D’s role in kidney health is complex. Its deficiency is both a consequence of kidney disease and a prognostic factor for progression of kidney damage and is linked to graft survival in kidney transplant recipients [42]. Vitamin D is also an extremely important therapeutic target, as its analogues have a role in the treatment of mineral and bone alterations, proteinuria, and in the reduction in kidney inflammation and fibrosis [54].

**Figure 2:**
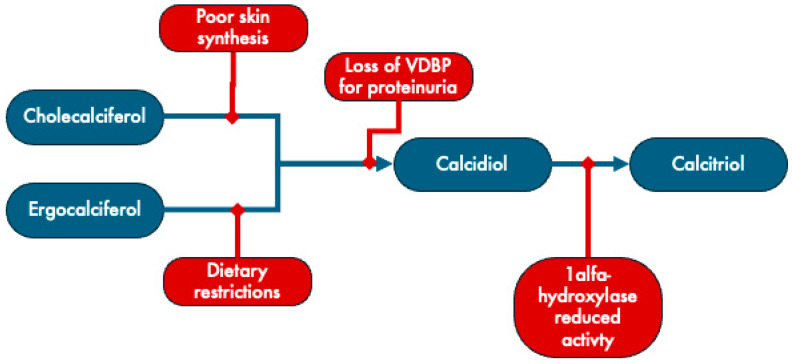
Mechanisms underlying vitamin D deficiency in CKD. VDBP: vitamin D binding protein. Adapted from Brandenburg [57].

Due to various factors, individuals with CKD frequently encounter deficiencies in both calcidiol and calcitriol (Figure 2). CKD hampers the activity of 1α-hydroxylase CYP27B1, the enzyme responsible for hydroxylation of calcidiol into calcitriol [55,56]. Moreover, this deficiency can result from compromised skin synthesis or dietary constraints, restricting the access to cholecalciferol and ergocalciferol precursors. Additionally, proteinuria and uremia associated with chronic kidney disease (CKD) can lead to the depletion of vitamin D binding proteins and 1,25-dihydroxyvitamin D [55].

### 3.1. Vitamin D in Mineral and Bone Disease

Vitamin D is a key component of calcium/phosphate homeostasis and bone metabolism: in healthy subjects, parathyroid hormone (PTH), fibroblast growth factor-23 (FGF23), and vitamin D act as deeply interlinked regulators of this delicate and complex physiological mechanism [58,59]. The disruptions in mineral metabolism caused by CKD, rising PTH, and lower vitamin D levels are presently recognized as integral components of the chronic kidney disease–mineral and bone disorder (CKD–MBD) definition [60].

Vitamin D exerts its effect on calcium homeostasis, forming a complex with VDR and RXR, binding to the vitamin D response element to regulate the transcription of various genes, including epithelial calcium channels and calcium-binding proteins [61,62,63]. Subsequently, calcitriol deficiency will result in reduced calcium absorption from the intestine; to counteract this effect and avoid hypocalcemia, PTHs activate osteoclasts, thus reabsorbing calcium from the bone. In CKD, various mechanisms contribute to the overproduction of PTH, a condition known as secondary hyperparathyroidism (sHPT), a disease totally different from disorders in the parathyroid glands (primary HPT) [57,58].

The clinical implications of CKD–MBD involve parathyroid gland hyperplasia, bone abnormalities, and vascular calcification; as CKD progresses, the parathyroid glands undergo nodular hyperplasia due to persistent overstimulation by hypocalcemia and hyperphosphoremia [58,62,64,65]. The reduced sensitivity to vitamin D and calcium signals, attributed to the loss of respective receptors, further complicates the situation, leading to parathyroidectomy in the most severe cases [60,62,66].

Bone abnormalities, encompassing different patterns under the term renal osteodystrophy, lead to osteoporosis and an increasing risk of fracture, worsening together with the decline in renal function [67].

The disturbance in mineral homeostasis within CKD–MBD, through the elevated serum phosphate levels leading to deposit of calcium phosphate salts in the arteries walls, heightens the risk of vascular calcification, thereby increasing susceptibility to cardiovascular diseases [66,68,69]. Managing mineral imbalances like hyperphosphatemia and SHPT is still regarded as one of the prevailing approaches for addressing vascular calcification in CKD. This involves the use of phosphate binders in hyperphosphatemic patients at all stages of CKD, along with implementing dietary phosphate restrictions and utilizing calcimimetics [70]. Vitamin D compounds continue to be one of the primary choices for preventing and treating SHPT in CKD [60].

### 3.2. Vitamin D as RAAS Inhibitor

**Figure 3:**
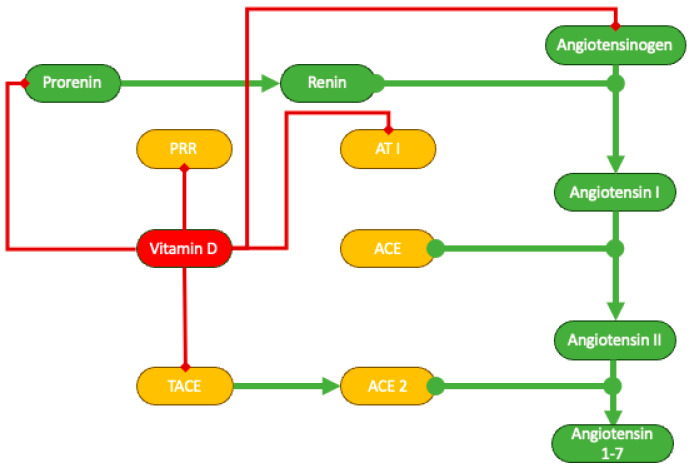
Schematic representation of vitamin D inhibition of RAAS. ACE, angiotensin-converting enzyme; AT1, angiotensin receptor type 1; PRR, prorenin receptor; TACE, tumor necrosis factor α-converting enzyme.

The role of vitamin D in renin–angiotensin–aldosterone system (RAAS) inhibition is nowadays undisputed [71,72]. In experimental models of chronic kidney disease, paricalcitol, a synthetic analogue of vitamin D, diminishes the renal expression of renin, the (pro)renin receptor, angiotensinogen, and the type 1 angiotensin receptor. Furthermore, vitamin D hinders the activity of tumor necrosis factor α-converting enzyme (TACE), which controls the shedding of angiotensin-converting enzyme 2 (ACE2), a crucial enzyme responsible for metabolizing angiotensin II in the proximal tubule (Figure 3) [72,73].

Several pioneering studies have found a negative correlation between the concentration of plasma 1,25(OH)2D3 and blood pressure, as well as plasma renin activity, in both normotensive men and individuals with essential hypertension [74,75,76]. It has been documented that supplementation with vitamin D3 reduces blood pressure in individuals with essential hypertension (19, 20). Treatment with 1,25(OH)2D3 also leads to a reduction in blood pressure, plasma renin activity, and angiotensin II levels in patients with hyperparathyroidism [77,78]. Furthermore, exposure to ultraviolet light, necessary for vitamin D biosynthesis, is inversely related to increases in blood pressure and the prevalence of hypertension in the general population, demonstrating blood pressure-lowering effects [79,80].

### 3.3. Vitamin D and Proteinuria

Proteinuria is one of the main predictors of chronic kidney disease progression and stands out as a potent and autonomous predictor of adverse outcomes in cardiovascular health. Importantly, these associations are significant regardless of the glomerular filtration rate level. Moreover, these connections hold true across populations with varying degrees of risk for kidney disease progression and cardiovascular disease development. The association between proteinuria and CVD persists even at proteinuria levels below existing thresholds for microalbuminuria [81,82]. Being recognized as the main therapeutic target in the management of CKD, it is not surprising that international guidelines recommend every possible effort to reduce it to the lowest achievable level [83].

Effective therapies that can reduce proteinuria include inhibitors of RAAS, such as angiotensin receptor blockers (ARBs) and angiotensin-converting enzyme inhibitors (ACEi) [84,85]. However, their effect is often suboptimal, and possible persisting residual proteinuria is still an important predictor of renal impairment. Since the acknowledgement of potential serious adverse effects of “dual blockade” of RAAS, combined with the lack of evidence of a reduction in mortality and improvement of kidney function of this therapeutic regimen, there has been a need for drugs capable of limiting residual proteinuria [86,87,88].

More recently, with the introduction of sodium–glucose transporter type 2 inhibitors (SGLT2i), a further step towards a better treatment of proteinuric patients has been made [89,90]. Notably, increasing evidence shows that SGLT2i can modulate phosphate homeostasis, increasing serum phosphate, PTH, and FGF23 [91,92]. Due to the role of FGF23 in promoting cardiac fibrosis [93], these data apparently contrast with the reduction in cardiovascular events with SGLT2i therapy [94]

Several studies reported vitamin D’s role in various groups of proteinuric patients: the exact mechanisms are still not fully understood, but they appear to be due to an inhibition of RAAS, as described earlier in this paper [95], and to a direct effect on podocytes. As they express both VDR and 1-α-hydroxylase, podocytes can produce calcitriol and respond to autocrine or endocrine calcitriol. In cultured podocytes, calcitriol triggers a dose-dependent activation of transcription of the nephrin gene [96,97]. Nephrin serves both structural and signaling functions, working in conjunction with other slit diaphragm components to create a permeable molecular sieve. This sieve primarily accounts for the retention of proteins [98,99].

Vitamin D analogues, such as paricalcitol, have shown a potential in treating residual proteinuria in various subsets of patients, including kidney transplant recipients [100,101,102,103]. Despite an increasing number of randomized controlled trial and observational studies, however, the quality of evidence and the strength of the recommendation are not yet able to suggest a routinary use of paricalcitol for the sole aim of reducing proteinuria, but further research is encouraged.

### 3.4. Anti-Inflammatory Effects

VDRs play a significant role in overseeing processes like inflammation, epithelial-to-mesenchymal transition, and podocyte integrity [104].

Together, VDR and vitamin D influence the apoptosis of cultured mouse podocytes and modulate transforming growth factor β (TGFβ) through the nuclear factor κB (NF-κB) pathway; VDR-mediated sequestration of NF-κB signaling also gives vitamin D potent antiproliferative, prodifferentiative, and immunomodulating activities, thus dampening renal inflammation [104,105].

Vitamin D also hinders NFκB transactivation by modulating the advanced glycation end products and their receptor (AGE–RAGE system), a mechanism underlying the progression of various kidney diseases, including diabetic nephropathy, hypertensive nephropathy, obesity-related glomerulopathy, lupus nephritis, amyloidosis, autosomal dominant polycystic kidney disease, and septic acute kidney injury [106,107,108]. It also promotes the production of IL-10 while reducing the production of TNF-α, IL-12, IL-6, and IFN-c, resulting in an anti-inflammatory cytokine profile [109]. Other research suggests that dendritic cells are the primary target of the immunosuppressive activity induced by vitamin D. This is because it hinders the differentiation, maturation, and survival of these cells, ultimately resulting in compromised activation of alloreactive T cells [110]. Moreover, many of the cells engaged in both innate (monocytes, dendritic cells) and adaptive (T cells, B cells) immune responses express both CYP27B1 and VDR. This suggests their ability to both synthesize calcitriol from calcidiol and respond to its effects through autocrine and paracrine pathways.

More recently, several studies have shown a great potential of vitamin D immunomodulatory activities in various non-renal immune diseases, such as vitiligo and multiple sclerosis [111,112,113].

The immunomodulatory effect of vitamin D, and especially its action on T cells, with the shift toward a less inflammatory and a more tolerogenic phenotype, could be responsible for its potential counteraction of chronic allograft dysfunction, thus enhancing graft survival [114,115,116,117,118]. Despite these considerations, data on the clinical effectiveness of vitamin D supplementation for prolonging graft survival are controversial; furthermore, much of the available evidence comes from observational studies rather than randomized controlled trials [40,119,120,121,122].

The anti-inflammatory and antiproliferative role of vitamin D also has important effects on atherosclerosis, as will be better described further in this paper.

## 4. Interplay between Vitamin D and Cardiovascular Disease

Cardiovascular disease stands as the predominant cause of global mortality and morbidity. Its multifaceted etiology involves an array of risk factors, categorized into modifiable biochemical or physiological characteristics—such as elevated blood pressure, increased plasma total cholesterol, hyperglycemia, obesity, or thrombogenic factors—and nonmodifiable personal characteristics, including age, sex, or a family history of coronary heart disease (CHD) or other atherosclerotic vascular diseases at an early age [123,124].

**Figure 4:**
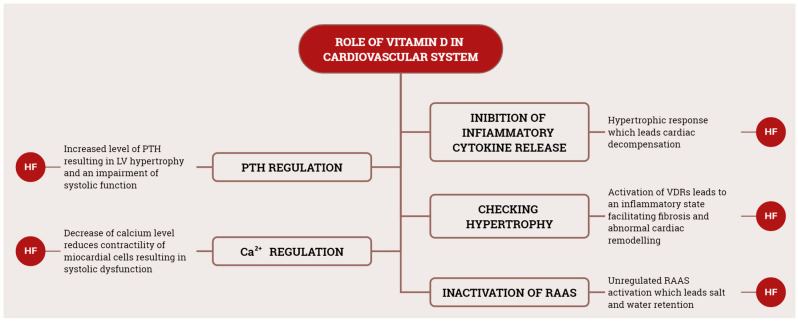
Schematic representation of main vitamin D roles in CVD. RAAS, renin–angiotensin–aldosterone system; HF, heart failure; PTH, parathyroid hormone.

Significant strides in scientific research have expanded our understanding of cardiovascular disease, uncovering novel therapeutic targets. Among these emerging targets, vitamin D has garnered attention for its potential role in the pathogenesis of various cardiovascular diseases [24]. Figure 4 provides a schematic summary of various roles of vitamin D in the genesis of CVD.

### 4.1. Hypertension

Untreated hypertension poses a significant risk for cardiovascular diseases like coronary artery atherosclerosis, stroke, or myocardial infarction [125,126]. Research findings indicate that a lack of vitamin D exacerbates the progression of hypertension (HT); thus, vitamin D deficiency emerges as an autonomous risk factor for elevated blood pressure and plays a role in fostering cardiovascular mortality [126,127,128].

Several mechanisms can explain vitamin D’s role in hypertension.

As previously stated, vitamin D exerts a regulatory activity in RAAS. These effects unsurprisingly show a consequence in the development of hypertension, as confirmed in many studies both on animal and human [129,130]. Vitamin D can reduce sympathetic activity directly related to high plasma levels of renin, which influences vascular tone through an increase in intraglomerular pressure [131].

Beyond that, vitamin D is involved in calcium homeostasis by increasing renal reabsorption, increasing calcium release from bone by osteoclasts, and stimulating the production of calcium transporters [132].

In addition, vitamin D acts at the level of peripheral vascular resistance tone by regulating the influx of calcium and thus acting on increased or decreased peripheral vascular resistance; in fact, VDR is also expressed on vascular smooth muscle cells, and directly influences muscle relaxation [132,133].

Vitamin D also seems to exert a direct impact on vascular stiffness. This is due to the presence of 1α-hydrolase in endothelial cells and vascular smooth muscle cells (VSMCs), enabling them to convert calcidiol to calcitriol [134]. Research indicates that inflammatory molecules like TNF-α and lipopolysaccharide activate this enzyme in Human Umbilical Vein Endothelial Cells (HUVECs) [135]. Furthermore, the addition of calcidiol and calcitriol externally attracts monocytes and increases their binding to HUVECs [135]. Vitamin D activation by macrophage is less tightly regulated than in the kidney; in atherosclerotic lesions, these macrophages penetrate the arterial wall, allowing the activated vitamin to directly influence VSMCs [136]. This can enhance the response to vasopressors, promote calcification, and induce cell dedifferentiation and oxidative stress [137,138,139,140].

Despite these proven effects, vitamin D supplementation has shown negligible effects in the treatment of hypertension in some recent clinical trials, although some studies are more encouraging. It is possible that our understanding of vitamin D’s effects on blood pressure regulation is still too poor to give us the ability to use it effectively in a clinical context [141].

### 4.2. Vitamin D Deficiency in Atherosclerosis

Atherosclerosis overwhelmingly stands as the predominant underlying factor for coronary artery, carotid, and peripheral arterial disease. This is a pathological condition characterized by changes in the wall of the arteries, which lose their elasticity due to the accumulation of calcium, cholesterol, inflammatory cells, and fibrotic material.

Among the many cardiovascular risk factors, an elevated plasma cholesterol level is probably unique in being sufficient to drive the development of atherosclerosis, even in the absence of other known risk factors [142,143,144].

Other risk factors involved in the atherosclerotic process include hypertension, male sex, diabetes mellitus, elevated homocysteine levels, and obesity. These factors contribute to accelerating the process of atherosclerosis triggered by lipoproteins [145].

Among these “classic”, well-established risk factors, several studies have shown possible involvement of vitamin D in the pathogenesis of atherosclerosis.

Vitamin D directly influences the cardiovascular system, as evidenced by the presence of VDRs in various cardiovascular cell types, including endothelial cells, vascular smooth muscle cells, circulating monocytes, platelets, dendritic cells, macrophages, and activated T lymphocytes [146].

Inside endothelial cells, vitamin D regulates the production of nitric oxide (NO) by modulating the activity of endothelial NO synthase (eNOS). In pathological situations, oxidative stress induced by the overproduction of reactive oxygen species (ROS) promotes NO breakdown and inhibits NO synthesis, leading to decreased NO availability. However, vitamin D counteracts the role of nicotinamide adenine dinucleotide phosphate (NADPH) oxidase, the generator of ROS, and augments antioxidant capacity by increasing the activity of antioxidant enzymes such as superoxide dismutase [147]. Studies have demonstrated that 1,25(OH)2D3 inhibits the proliferative effects of epidermal growth factor and endothelin on vascular smooth muscle cells (VSMCs). Specifically, it achieves the latter by reducing the activity of cyclin-dependent kinase 2, a regulator of the cell cycle machinery [148]. The impact of 1α,25(OH)2D3 on VSMC migration seems to vary. At elevated levels, calcitriol can stimulate VSMC migration. Conversely, at physiological concentrations, both 25(OH)D and calcitriol impede VSMC migration and proliferation by decreasing the activity of vitamin D-binding protein. This effect is mediated by the reduction in extracellular signal-regulated kinase 1/2 phosphorylation [149]. The decrease in the formation of atherosclerotic lesions resulted from the inhibition of immune responses, wherein at least two types of cells play a crucial role in the effects of vitamin D3 (specifically, CD4+CD25+ Forkhead box protein [Foxp] 3+ regulatory T cells [Tregs] and dendritic cells [DCs]) [150]. Additionally, there exists a putative role for vitamin D in the process of vascular calcification [151]. 1,25-vitamin D has a significant association with vascular calcification, and, quite unexpectedly, it is a negative correlation, revealing that higher serum levels of 1,25-vitamin D were associated with less vascular calcification. Vitamin D, in addition to being involved in calcium deposition in the axillary skeleton, could in fact also regulate calcium deposition in the vascular wall [151].

### 4.3. The Role of Vitamin D in Heart Failure

Heart failure (HF) is a pathological state characterized by the heart’s inability to meet the metabolic demands of the body. The prevalence of HF varies significantly, ranging from 1% to 12%, as documented in comprehensive reports from the United States and Europe [152]. At the core of HF pathology lies the breakdown of compensatory mechanisms designed to ensure sufficient nutrient delivery to tissues. These mechanisms encompass the neurohormonal system, renin–angiotensin system, aldosterone, parietal remodeling, and chronic inflammation [153].

Patients with HF exhibiting low vitamin D levels tend to experience unfavorable outcomes, aligning with established clinical correlations and biomarkers [154]. In the context of HF affecting myocardial cells, the surplus of ionized calcium (Ca2) detrimentally impacts the contraction and relaxation of the heart [155]. Conversely, vitamin D deficiency may perturb the activities of Ca2 in cardiac cells, contributing to fibrosis, intra-organizational inflammation, and cardiomyocyte hypertrophy [156,157]. Additionally, diminished vitamin D levels can induce inflammation, activate the renin–angiotensin system, and lead to endothelial dysfunction [158].

Several epidemiological and observational studies confirmed a higher risk of cardiovascular events and related mortality in patients with vitamin D deficiency [159,160]; furthermore, this category of individuals shows significantly higher LV wall thickness, diameter, and LV mass, and impaired myocardial performance index in comparison to the rest of the population [161,162].

While observational and epidemiological data, together with pathophysiological studies, suggest that vitamin D supplementation may ameliorate ventricular remodeling in HF patients, the clarity of this relationship remains elusive [163].

Evidence from many interventional studies, such as RECORD, EVITA, ViDA, VINDICATE, and the most recent VITAL, has shown little or no benefit from vitamin D supplementation in reducing adverse cardiovascular events or CVD-related mortality [164,165,166,167,168].

### 4.4. Atrial Fibrillation

Atrial fibrillation (AF), the most prevalent sustained arrhythmia, is linked to substantial morbidity, diminished functional status, compromised quality of life, and heightened mortality, with an adjusted rate of 4.72% per year. A significant proportion of deaths, approximately 46%, are attributed to cardiological causes, encompassing sudden cardiac death, heart failure, and myocardial infarction. In contrast, a minority are associated with nonhemorrhagic strokes (5.7%) or hemorrhagic events (5.6%) [169].

The established risk factors for AF include advanced age, male sex, hypertension, alcohol consumption, and valvular disease, with emerging factors such as hypertrophic cardiomyopathy, obstructive sleep apnea syndrome (OSAS), coronary artery disease, and chronic kidney disease gaining recognition [170,171]. The role of vitamin D in the pathogenesis of atrial fibrillation remains contentious, with divergent findings in the literature. Some studies indicate a positive correlation between hypovitaminosis D and atrial fibrillation, while others do not establish a clear link [172,173]. A plausible correlation may lie in vitamin D’s interference with reactive oxygen species (ROS) production in the atrium, contributing to the arrhythmic substrate of atrial fibrillation. Additionally, vitamin D has been observed to negatively modulate the renin–angiotensin–aldosterone system, thereby mitigating atrial remodeling, a phenomenon commonly observed in atrial fibrillation [174].

### 4.5. Vitamin D, Cardiac Fibrosis, and Cardiorenal Syndrome

As previously stated, CKD progression triggers a gradual increase in FGF23 levels, in an attempt to overcome the impaired phosphate metabolism [175], binding to FGF receptor (FGFR) via an associated coreceptor, klotho [176]. However, FGF23 has a putative role in promoting cardiac fibrosis and left ventricular hypertrophy, as shown in animal and human studies, via FGFR-dependent activation of the calcineurin–NFAT pathway; this mechanism appears to be independent by the presence of klotho, which is necessary for FGF23 in exerting its role in parathyroid glands and kidneys [177,178]. Conversely, other studies suggest that the soluble form of klotho (s-klotho) can prevent these effects of FGF23 on cardiomyocytes.

This FGF23-mediated disruption of cardiac tissue and the subsequent left ventricular hypertrophy establish one of the many forms of the cardiorenal syndrome [179].

Several studies enquired about the efficacy of vitamin D and its analogues in contrasting cardiac fibrosis due to high levels of FGF23: while animal models showed promising results with the use of vitamin D analogues such as paricalcitol [180,181], data derived from clinical settings are ambiguous [182,183]. Even further, the most recent clinical trials showed no cardiovascular benefit in vitamin D supplementation [184].

It must be noted that in some genetic forms of hypophosphatemia and rickets characterized by increased levels of FGF23, such as X-linked hypophosphatemia, cardiac abnormalities are not a common finding: this opens up controversies on the effective role of FGF23 in developing cardiac fibrosis and left ventricular hypertrophy [185].

## 5. Vitamin D in Pharmacological Therapy

Vitamin D serum essays and supplement prescription are becoming increasingly common worldwide, especially in the last decade; despite this great interest both by physicians and patients towards this issue, inappropriate testing can be confounding, and inadequate prescription can lead to potentially harmful consequences, not to mention the associated costs for individuals and society [186].

To this day, vitamin D supplementation is recommended by numerous scientific societies and experts panels, mainly in the treatment of osteoporosis, CKD–mineral bone disorder, and prevention of rickets [30,31,60]; these recommendations, however, are not homogeneous, but rather contradictory, showing that the scientific debate is still ongoing due to contrasting evidence.

An increasing amount of data, as described earlier, suggest a potential pathogenetic role for vitamin D deficiency in a vast number of diseases and conditions; thus, it is unsurprising that the idea of supplementing vitamin D, both as a nutraceutical supplement in the form of ergocalciferol, or as cholecalciferol, can be fascinating for both healthcare providers and patients. During the early phases of the COVID-19 pandemic, vitamin D was also enthusiastically proposed for the treatment of SARS-CoV2 infection due to previous evidence of a protective effect against respiratory tract infections [187], but further evidence did not support this hypothesis [188,189].

Many observational studies suggest that vitamin D supplementation can, in fact, provide a beneficial effect in various settings, such as cardiovascular disease, multiple myeloma and solid tumors, multiple sclerosis, rheumatoid arthritis, and other autoimmune diseases; however, high-quality evidence is still lacking, and there is still no clear indication for the use of vitamin D in any of the abovementioned diseases [190,191,192,193]. Even for the most well-established indications, such as the treatment of osteoporosis and fracture patients, evidence is uncertain and the consensus is lacking [194,195,196,197].

Furthermore, several randomized control trials failed to demonstrate a significant benefit from vitamin D supplementation [198,199,200,201,202], deflating the enthusiasm for its use in routinary clinical context.

### Vitamin D Toxicity

Despite being often perceived as innocuous, inappropriate vitamin D assumption can lead to potential harm. As an example, massive amounts of vitamin D are currently used as rodenticide in pest control [203]. The great availability of over-the-counter supplements and unthoughtful prescription by some physicians are causing an increase in vitamin D intoxications [204].

The classical clinical presentation of vitamin D toxicity includes some characteristics signs, such as soft tissue calcification, hypercalcemia, and hypercalciuria, and it is usually related to a chronic oral assumption of more than 250 µg of vitamin D [205]. Symptoms may vary, ranging from polyuria and thirst to more severe and potentially life-threatening neurological manifestations such as confusion, seizures, and coma. Other frequently associated symptoms include abdominal pain, polydipsia, pancreatitis, bradyarrhythmias, vascular calcifications, nephrocalcinosis, and acute kidney injury (AKI) [206,207,208,209]. In the most severe cases, patients with vitamin D toxicity can even require dialysis, both for the treatment of incident AKI, for chronic kidney disease due to nephrocalcinosis, or to lower serum calcium levels [206].

Furthermore, in kidney stone formers, a relatively mild hypercalcemia and hypercalciuria increase stone formation, and vitamin D use should be avoided if not otherwise deemed necessary [210,211].

In conclusion, the use of vitamin D in pharmacological therapy cannot be routinary and must be carefully evaluated, due to both a lack of evidence of potential benefits and to the presence of potential harmful effects.

## 6. Conclusions

Over the decades, since the discovery of its deficiency disease by Casimir Funk [212], vitamin D has captured the attention of scientists from all around the world. This led to the acknowledgement of its various pleiotropic effects, ranging from anti-infective effects, reduction in metabolic complications, to cancer prevention, and, as extensively described, in kidney and cardiovascular health. However, recent findings from randomized clinical trials and meta-analyses have tempered the enthusiasm surrounding the purported “pleiotropic” effects of vitamin D [213]. This is because there is a lack of clear evidence demonstrating the beneficial effects of vitamin D supplementation across various clinical scenarios [129,198,199,200,201,214,215]. On the other hand, inappropriate vitamin D supplementation can lead to serious, although rare, health issues, mainly linked to hypercalcemia [208,209,216].

In conclusion, while the pleiotropic effects of vitamin D on kidney and cardiovascular health have been extensively explored, it is essential to acknowledge that conclusive evidence regarding its clinical efficacy is still lacking. Despite numerous studies, the intricate interplay between vitamin D and these health outcomes requires many more years of intensive research for a comprehensive understanding. Some of the limitations of available studies on vitamin D can explain the confusion generated by different and sometimes contrasting findings. These limitations often include a poor selection of the subjects enrolled, lack of a clear and universally shared definition for vitamin D deficiency itself, the many different laboratory essays used in both forms of vitamin D assessment, and a still incomplete comprehension of the pathophysiology balancing calcidiol and calcitriol serum levels. Thus, there is great need in understanding and clarifying these topics, in order to obtain a more solid ground of knowledge that can be used to design more effective clinical studies.

Furthermore, there is evidence indicating that vitamin D levels serve as a reflection of a generally healthy lifestyle, more than being an etiologic factor for various diseases. It has been observed that inadequate or deficient levels of vitamin D often correlate with unhealthy and sedentary lifestyles, thereby posing a risk for negative health consequences [217]. This important consideration can probably change the way we are currently investigating vitamin D’s role in health and diseases.

The journey towards unraveling the true impact of vitamin D on kidney and cardiovascular health remains a complex and evolving path, emphasizing the need for continued scientific exploration in this field.

## Data Availability

Not applicable.

## References

[1] Bikbov B., Purcell C.A., Levey A.S., Smith M., Abdoli A., Abebe M., Adebayo O.M., Afarideh M., Agarwal S.K., Agudelo-Botero M. (2020). Global, Regional, and National Burden of Chronic Kidney Disease, 1990–2017: A Systematic Analysis for the Global Burden of Disease Study 2017. Lancet.

[2] Foreman K.J., Marquez N., Dolgert A., Fukutaki K., Fullman N., McGaughey M., Pletcher M.A., Smith A.E., Tang K., Yuan C.-W. (2018). Forecasting Life Expectancy, Years of Life Lost, and All-Cause and Cause-Specific Mortality for 250 Causes of Death: Reference and Alternative Scenarios for 2016–40 for 195 Countries and Territories. Lancet.

[3] Mensah G.A., Wei G.S., Sorlie P.D., Fine L.J., Rosenberg Y., Kaufmann P.G., Mussolino M.E., Hsu L.L., Addou E., Engelgau M.M. (2017). Decline in Cardiovascular Mortality: Possible Causes and Implications. Circ. Res..

[4] Webster A.C., Nagler E.V., Morton R.L., Masson P. (2017). Chronic Kidney Disease. Lancet.

[5] Francula-Zaninovic S., Nola I.A. (2018). Management of Measurable Variable Cardiovascular Disease’ Risk Factors. CCR.

[6] Di Pietro P., Izzo C., Carrizzo A. (2023). Editorial: The Role of Metabolic Syndrome and Disorders in Cardiovascular Disease. Front. Endocrinol..

[7] Cirillo M., Bilancio G., Secondulfo C., Iesce G., Ferrara C., Terradura-Vagnarelli O., Laurenzi M. (2022). Relation of Alcohol Intake to Kidney Function and Mortality Observational, Population-Based, Cohort Study. Nutrients.

[8] Izzo M., Carrizzo A., Izzo C., Cappello E., Cecere D., Ciccarelli M., Iannece P., Damato A., Vecchione C., Pompeo F. (2021). Vitamin D: Not Just Bone Metabolism but a Key Player in Cardiovascular Diseases. Life.

[9] Matsushita K., Ballew S.H., Wang A.Y.-M., Kalyesubula R., Schaeffner E., Agarwal R. (2022). Epidemiology and Risk of Cardiovascular Disease in Populations with Chronic Kidney Disease. Nat. Rev. Nephrol..

[10] Bilancio G., Celano M., Cozza V., Zingone F., Palladino G., Cirillo M. (2017). Early Prediction of Cardiovascular Disease in Kidney Transplant Recipients. Transplant. Proc..

[11] Jankowski J., Floege J., Fliser D., Böhm M., Marx N. (2021). Cardiovascular Disease in Chronic Kidney Disease: Pathophysiological Insights and Therapeutic Options. Circulation.

[12] Sarnak M.J., Levey A.S., Schoolwerth A.C., Coresh J., Culleton B., Hamm L.L., McCullough P.A., Kasiske B.L., Kelepouris E., Klag M.J. (2003). Kidney Disease as a Risk Factor for Development of Cardiovascular Disease: A Statement From the American Heart Association Councils on Kidney in Cardiovascular Disease, High Blood Pressure Research, Clinical Cardiology, and Epidemiology and Prevention. Circulation.

[13] Bover J., Evenepoel P., Urena-Torres P., Vervloet M.G., Brandenburg V., Mazzaferro S., Covic A., Goldsmith D., Massy Z.A., Cozzolino M. (2015). Pro: Cardiovascular Calcifications Are Clinically Relevant. Nephrol. Dial. Transplant..

[14] Chen J., Budoff M.J., Reilly M.P., Yang W., Rosas S.E., Rahman M., Zhang X., Roy J.A., Lustigova E., Nessel L. (2017). Coronary Artery Calcification and Risk of Cardiovascular Disease and Death Among Patients With Chronic Kidney Disease. JAMA Cardiol..

[15] Carrizzo A., Izzo C., Forte M., Sommella E., Di Pietro P., Venturini E., Ciccarelli M., Galasso G., Rubattu S., Campiglia P. (2020). A Novel Promising Frontier for Human Health: The Beneficial Effects of Nutraceuticals in Cardiovascular Diseases. Int. J. Mol. Sci..

[16] Izzo C., Secondulfo C., Bilancio G., Visco V., Virtuoso N., Migliarino S., Ciccarelli M., Di Pietro P., La Mura L., Damato A. (2024). Chronic Kidney Disease with Mineral Bone Disorder and Vascular Calcification: An Overview. Life.

[17] Danik J.S., Manson J.E. (2012). Vitamin D and Cardiovascular Disease. Curr. Treat. Options Cardiovasc. Med..

[18] Lavie C.J., Lee J.H., Milani R.V. (2011). Vitamin D and Cardiovascular Disease. J. Am. Coll. Cardiol..

[19] Demer L.L., Hsu J.J., Tintut Y. (2018). Steroid Hormone Vitamin D: Implications for Cardiovascular Disease. Circ. Res..

[20] Romano M.R., Biagioni F., Carrizzo A., Lorusso M., Spadaro A., Micelli Ferrari T., Vecchione C., Zurria M., Marrazzo G., Mascio G. (2014). Effects of Vitamin B12 on the Corneal Nerve Regeneration in Rats. Exp. Eye Res..

[21] Vieth R. (2020). Vitamin D Supplementation: Cholecalciferol, Calcifediol, and Calcitriol. Eur. J. Clin. Nutr..

[22] Vieth R., Chan P.-C.R., MacFarlane G.D. (2001). Efficacy and Safety of Vitamin D3 Intake Exceeding the Lowest Observed Adverse Effect Level. Am. J. Clin. Nutr..

[23] Bikle D.D. (2014). Vitamin D Metabolism, Mechanism of Action, and Clinical Applications. Chem. Biol..

[24] Holick M.F. (2007). Vitamin D Deficiency. N. Engl. J. Med..

[25] Holick M.F. (2005). Vitamin D in Health and Disease: Vitamin D for Health and in Chronic Kidney Disease. Semin. Dial..

[26] Pike J.W., Meyer M.B. (2010). The Vitamin D Receptor: New Paradigms for the Regulation of Gene Expression by 1,25-Dihydroxyvitamin D3. Endocrinol. Metab. Clin. North Am..

[27] Stivelman E., Retnakaran R. (2012). Role of Vitamin D in the Pathophysiology and Treatment of Type 2 Diabetes. CDR.

[28] Ellison T.I., Dowd D.R., MacDonald P.N. (2005). Calmodulin-Dependent Kinase IV Stimulates Vitamin D Receptor-Mediated Transcription. Mol. Endocrinol..

[29] Latic N., Erben R.G. (2020). Vitamin D and Cardiovascular Disease, with Emphasis on Hypertension, Atherosclerosis, and Heart Failure. Int. J. Mol. Sci..

[30] Holick M.F., Binkley N.C., Bischoff-Ferrari H.A., Gordon C.M., Hanley D.A., Heaney R.P., Murad M.H., Weaver C.M. (2011). Evaluation, Treatment, and Prevention of Vitamin D Deficiency: An Endocrine Society Clinical Practice Guideline. J. Clin. Endocrinol. Metab..

[31] (2011). Dietary Reference Intakes for Calcium and Vitamin D.

[32] Munns C.F., Shaw N., Kiely M., Specker B.L., Thacher T.D., Ozono K., Michigami T., Tiosano D., Mughal M.Z., Mäkitie O. (2016). Global Consensus Recommendations on Prevention and Management of Nutritional Rickets. J. Clin. Endocrinol. Metab..

[33] EFSA Panel on Dietetic Products, Nutrition and Allergies (NDA) (2016). Dietary Reference Values for Vitamin D. EFSA J..

[34] Jacobs E.T., Martínez M.E., Jurutka P.W. (2011). Vitamin D: Marker or Mechanism of Action?. Cancer Epidemiol. Biomark. Prev..

[35] Cirillo M., Bilancio G., Guarino E., Cavallo P., Lombardi C., Costanzo S., De Curtis A., Di Castelnuovo A., Iacoviello L. (2019). Vitamin D Status and Indices of Mineral Homeostasis in the Population: Differences Between 25-Hydroxyvitamin D and 1,25-Dihydroxyvitamin D. Nutrients.

[36] Cashman K.D. (2020). Vitamin D Deficiency: Defining, Prevalence, Causes, and Strategies of Addressing. Calcif. Tissue Int..

[37] Cashman K.D., Dowling K.G., Škrabáková Z., Gonzalez-Gross M., Valtueña J., De Henauw S., Moreno L., Damsgaard C.T., Michaelsen K.F., Mølgaard C. (2016). Vitamin D Deficiency in Europe: Pandemic?. Am. J. Clin. Nutr..

[38] Ciacci C., Bilancio G., Russo I., Iovino P., Cavallo P., Santonicola A., Bucci C., Cirillo M., Zingone F. (2020). 25-Hydroxyvitamin D, 1,25-Dihydroxyvitamin D, and Peripheral Bone Densitometry in Adults with Celiac Disease. Nutrients.

[39] Cirillo M., Bilancio G., Cavallo P., Costanzo S., De Curtis A., Di Castelnuovo A., Iacoviello L. (2022). Correlates of Calcidiol Deficiency in Adults-Cross-Sectional, Observational, Population-Based Study. Nutrients.

[40] Courbebaisse M., Alberti C., Colas S., Prié D., Souberbielle J.-C., Treluyer J.-M., Thervet E. (2014). VITamin D Supplementation in renAL Transplant Recipients (VITALE): A Prospective, Multicentre, Double-Blind, Randomized Trial of Vitamin D Estimating the Benefit and Safety of Vitamin D3 Treatment at a Dose of 100,000 UI Compared with a Dose of 12,000 UI in Renal Transplant Recipients: Study Protocol for a Double-Blind, Randomized, Controlled Trial. Trials.

[41] Zhou Q., Li L., Chen Y., Zhang J., Zhong L., Peng Z., Xing T. (2019). Vitamin D Supplementation Could Reduce the Risk of Acute Cellular Rejection and Infection in Vitamin D Deficient Liver Allograft Recipients. Int. Immunopharmacol..

[42] Messa P., Regalia A., Alfieri C. (2017). Nutritional Vitamin D in Renal Transplant Patients: Speculations and Reality. Nutrients.

[43] Binkley N., Krueger D., Lensmeyer G. (2009). 25-Hydroxyvitamin D Measurement, 2009: A Review for Clinicians. J. Clin. Densitom..

[44] Farrell C.-J.L., Martin S., McWhinney B., Straub I., Williams P., Herrmann M. (2012). State-of-the-Art Vitamin D Assays: A Comparison of Automated Immunoassays with Liquid Chromatography–Tandem Mass Spectrometry Methods. Clin. Chem..

[45] Fraser W.D., Milan A.M. (2013). Vitamin D Assays: Past and Present Debates, Difficulties, and Developments. Calcif. Tissue Int..

[46] Freedman B.I., Register T.C. (2012). Effect of Race and Genetics on Vitamin D Metabolism, Bone and Vascular Health. Nat. Rev. Nephrol..

[47] Levin G.P., Robinson-Cohen C., De Boer I.H., Houston D.K., Lohman K., Liu Y., Kritchevsky S.B., Cauley J.A., Tanaka T., Ferrucci L. (2012). Genetic Variants and Associations of 25-Hydroxyvitamin D Concentrations With Major Clinical Outcomes. JAMA.

[48] Brodie A.M., Lucas R.M., Harrison S.L., Van Der Mei I.A.F., Armstrong B., Kricker A., Mason R.S., McMichael A.J., Nowak M., Whiteman D.C. (2013). The AusD Study: A Population-Based Study of the Determinants of Serum 25-Hydroxyvitamin D Concentration Across a Broad Latitude Range. Am. J. Epidemiol..

[49] Powe C.E., Evans M.K., Wenger J., Zonderman A.B., Berg A.H., Nalls M., Tamez H., Zhang D., Bhan I., Karumanchi S.A. (2013). Vitamin D–Binding Protein and Vitamin D Status of Black Americans and White Americans. N. Engl. J. Med..

[50] Denburg M.R., Hoofnagle A.N., Sayed S., Gupta J., De Boer I.H., Appel L.J., Durazo-Arvizu R., Whitehead K., Feldman H.I., Leonard M.B. (2016). Comparison of Two ELISA Methods and Mass Spectrometry for Measurement of Vitamin D-Binding Protein: Implications for the Assessment of Bioavailable Vitamin D Concentrations Across Genotypes. J. Bone Miner. Res..

[51] Bikle D., Bouillon R., Thadhani R., Schoenmakers I. (2017). Vitamin D Metabolites in Captivity? Should We Measure Free or Total 25(OH)D to Assess Vitamin D Status?. J. Steroid Biochem. Mol. Biol..

[52] Franca Gois P.H., Wolley M., Ranganathan D., Seguro A.C. (2018). Vitamin D Deficiency in Chronic Kidney Disease: Recent Evidence and Controversies. Int. J. Environ. Res. Public Health.

[53] Li M., Li Y. (2020). Prevalence and Influencing Factors of Vitamin D Deficiency in Chronic Kidney Disease: A Cross-Sectional Study. Int. J. Clin. Pharmacol. Ther..

[54] Yang L., Ma J., Zhang X., Fan Y., Wang L. (2012). Protective Role of the Vitamin D Receptor. Cell. Immunol..

[55] Nigwekar S.U., Tamez H., Thadhani R.I. (2014). Vitamin D and Chronic Kidney Disease–Mineral Bone Disease (CKD–MBD). Bonekey Rep..

[56] Zhou S., Glowacki J. (2017). Chronic Kidney Disease and Vitamin D Metabolism in Human Bone Marrow–Derived MSCs. Ann. N. Y. Acad. Sci..

[57] Brandenburg V., Ketteler M. (2022). Vitamin D and Secondary Hyperparathyroidism in Chronic Kidney Disease: A Critical Appraisal of the Past, Present, and the Future. Nutrients.

[58] Fusaro M., Pereira L., Bover J. (2023). Current and Emerging Markers and Tools Used in the Diagnosis and Management of Chronic Kidney Disease–Mineral and Bone Disorder in Non-Dialysis Adult Patients. J. Clin. Med..

[59] Pascale A.V., Finelli R., Giannotti R., Visco V., Fabbricatore D., Matula I., Mazzeo P., Ragosa N., Massari A., Izzo R. (2018). Vitamin D, Parathyroid Hormone and Cardiovascular Risk: The Good, the Bad and the Ugly. J. Cardiovasc. Med..

[60] Kidney Disease: Improving Global Outcomes (KDIGO) CKD-MBD Update Work Group (2017). KDIGO 2017 Clinical Practice Guideline Update for the Diagnosis, Evaluation, Prevention, and Treatment of Chronic Kidney Disease–Mineral and Bone Disorder (CKD-MBD). Kidney Int. Suppl..

[61] Galuška D., Pácal L., Kaňková K. (2021). Pathophysiological Implication of Vitamin D in Diabetic Kidney Disease. Kidney Blood Press Res..

[62] Cunningham J., Locatelli F., Rodriguez M. (2011). Secondary Hyperparathyroidism: Pathogenesis, Disease Progression, and Therapeutic Options. Clin. J. Am. Soc. Nephrol..

[63] Christensen M.H.E., Apalset E.M., Nordbø Y., Varhaug J.E., Mellgren G., Lien E.A. (2013). 1,25-Dihydroxyvitamin D and the Vitamin D Receptor Gene Polymorphism Apa1 Influence Bone Mineral Density in Primary Hyperparathyroidism. PLoS ONE.

[64] Drueke T., Martin D., Rodriguez M. (2007). Can Calcimimetics Inhibit Parathyroid Hyperplasia? Evidence from Preclinical Studies. Nephrol. Dial. Transpl..

[65] Rodriguez M., Nemeth E., Martin D. (2005). The Calcium-Sensing Receptor: A Key Factor in the Pathogenesis of Secondary Hyperparathyroidism. Am. J. Physiol.-Ren. Physiol..

[66] Ketteler M., Bover J., Mazzaferro S. (2023). Treatment of Secondary Hyperparathyroidism in Non-Dialysis CKD: An Appraisal 2022s. Nephrol. Dial. Transplant..

[67] Naylor K.L., McArthur E., Leslie W.D., Fraser L.-A., Jamal S.A., Cadarette S.M., Pouget J.G., Lok C.E., Hodsman A.B., Adachi J.D. (2014). The Three-Year Incidence of Fracture in Chronic Kidney Disease. Kidney Int..

[68] Yamada S., Giachelli C.M. (2017). Vascular Calcification in CKD-MBD: Roles for Phosphate, FGF23, and Klotho. Bone.

[69] Cirillo C., Bilancio G., Natale F., Concilio C., Russo M.G., Calabrò P., Cirillo M. (2017). Cardiovascular Calcification and Subcortical Bone Demineralization in Hypertension. Hypertens. Res..

[70] Reiss A.B., Miyawaki N., Moon J., Kasselman L.J., Voloshyna I., D’Avino R., De Leon J. (2018). CKD, Arterial Calcification, Atherosclerosis and Bone Health: Inter-Relationships and Controversies. Atherosclerosis.

[71] Li Y.C., Kong J., Wei M., Chen Z.-F., Liu S.Q., Cao L.-P. (2002). 1,25-Dihydroxyvitamin D3 Is a Negative Endocrine Regulator of the Renin-Angiotensin System. J. Clin. Investig..

[72] Freundlich M., Quiroz Y., Zhang Z., Zhang Y., Bravo Y., Weisinger J.R., Li Y.C., Rodriguez-Iturbe B. (2008). Suppression of Renin–Angiotensin Gene Expression in the Kidney by Paricalcitol. Kidney Int..

[73] Dusso A., Arcidiacono M.V., Yang J., Tokumoto M. (2010). Vitamin D Inhibition of TACE and Prevention of Renal Osteodystrophy and Cardiovascular Mortality. J. Steroid Biochem. Mol. Biol..

[74] Lind L., Hänni A., Lithell H., Hvarfner A., Sörensen O.H., Ljunghall S. (1995). Vitamin D Is Related to Blood Pressure and Other Cardiovascular Risk Factors in Middle-Aged Men. Am J Hypertens.

[75] Resnick L.M. (1986). Calcium-Regulating Hormones in Essential Hypertension: Relation to Plasma Renin Activity and Sodium Metabolism. Ann. Intern. Med..

[76] Kristal-Boneh E., Froom P., Harari G., Ribak J. (1997). Association of Calcitriol and Blood Pressure in Normotensive Men. Hypertension.

[77] Kimura Y., Kawamura M., Owada M., Oshima T., Murooka M., Fujiwara T., Hiramori K. (1999). Effectiveness of 1,25-Dihydroxyvitamin D Supplementation on Blood Pressure Reduction in a Pseudohypoparathyroidism Patient with High Renin Activity. Intern. Med..

[78] Park C.W., Oh Y.S., Shin Y.S., Kim C.-M., Kim Y.-S., Kim S.Y., Choi E.J., Chang Y.S., Bang B.K. (1999). Intravenous Calcitriol Regresses Myocardial Hypertrophy in Hemodialysis Patients with Secondary Hyperparathyroidism. Am. J. Kidney Dis..

[79] Krause R., Bühring M., Hopfenmüller W., Holick M.F., Sharma A.M. (1998). Ultraviolet B and Blood Pressure. Lancet.

[80] Weber K.T., Rosenberg E.W., Sayre R.M. (2004). Suberythemal Ultraviolet Exposure and Reduction in Blood Pressure. Am. J. Med..

[81] Iseki K. (2013). Proteinuria as a Predictor of Rapid eGFR Decline. Nat. Rev. Nephrol..

[82] Sarnak M.J., Astor B.C. (2011). Implications of Proteinuria: CKD Progression and Cardiovascular Outcomes. Adv. Chronic Kidney Dis..

[83] (2013). Summary of Recommendation Statements. Kidney Int. Suppl..

[84] Brenner B.M., Cooper M.E., de Zeeuw D., Keane W.F., Mitch W.E., Parving H.H., Remuzzi G., Snapinn S.M., Zhang Z., Shahinfar S. (2001). Effects of Losartan on Renal and Cardiovascular Outcomes in Patients with Type 2 Diabetes and Nephropathy. N. Engl. J. Med..

[85] Lewis E.J., Hunsicker L.G., Clarke W.R., Berl T., Pohl M.A., Lewis J.B., Ritz E., Atkins R.C., Rohde R., Raz I. (2001). Renoprotective Effect of the Angiotensin-Receptor Antagonist Irbesartan in Patients with Nephropathy Due to Type 2 Diabetes. N. Engl. J. Med..

[86] Makani H., Bangalore S., Desouza K.A., Shah A., Messerli F.H. (2013). Efficacy and Safety of Dual Blockade of the Renin-Angiotensin System: Meta-Analysis of Randomised Trials. BMJ.

[87] Fernandez Juarez G., Luño J., Barrio V., De Vinuesa S.G., Praga M., Goicoechea M., Cachofeiro V., Nieto J., Fernández Vega F., Tato A. (2013). Effect of Dual Blockade of the Renin-Angiotensin System on the Progression of Type 2 Diabetic Nephropathy: A Randomized Trial. Am. J. Kidney Dis..

[88] Parving H.-H., Brenner B.M., McMurray J.J.V., De Zeeuw D., Haffner S.M., Solomon S.D., Chaturvedi N., Persson F., Desai A.S., Nicolaides M. (2012). Cardiorenal End Points in a Trial of Aliskiren for Type 2 Diabetes. N. Engl. J. Med..

[89] The EMPA-KIDNEY Collaborative Group (2023). Empagliflozin in Patients with Chronic Kidney Disease. N. Engl. J. Med..

[90] Heerspink H.J.L., Stefánsson B.V., Correa-Rotter R., Chertow G.M., Greene T., Hou F.-F., Mann J.F.E., McMurray J.J.V., Lindberg M., Rossing P. (2020). Dapagliflozin in Patients with Chronic Kidney Disease. N. Engl. J. Med..

[91] de Jong M.A., Petrykiv S.I., Laverman G.D., van Herwaarden A.E., de Zeeuw D., Bakker S.J.L., Heerspink H.J.L., de Borst M.H. (2019). Effects of Dapagliflozin on Circulating Markers of Phosphate Homeostasis. Clin. J. Am. Soc. Nephrol..

[92] Blau J.E., Bauman V., Conway E.M., Piaggi P., Walter M.F., Wright E.C., Bernstein S., Courville A.B., Collins M.T., Rother K.I. (2018). Canagliflozin Triggers the FGF23/1,25-Dihydroxyvitamin D/PTH Axis in Healthy Volunteers in a Randomized Crossover Study. JCI Insight.

[93] Murray S.L., Wolf M. (2022). Exercising the FGF23-Cardiac Axis. Kidney360.

[94] Wiviott S.D., Raz I., Bonaca M.P., Mosenzon O., Kato E.T., Cahn A., Silverman M.G., Zelniker T.A., Kuder J.F., Murphy S.A. (2019). Dapagliflozin and Cardiovascular Outcomes in Type 2 Diabetes. N. Engl. J. Med..

[95] Pérez-Gómez M.V., Ortiz-Arduán A., Lorenzo-Sellares V. (2013). Vitamina D y proteinuria: Revisión crítica de las bases moleculares y de la experiencia clínica. Nefrología.

[96] Matsui I., Hamano T., Tomida K., Inoue K., Takabatake Y., Nagasawa Y., Kawada N., Ito T., Kawachi H., Rakugi H. (2009). Active Vitamin D and Its Analogue, 22-Oxacalcitriol, Ameliorate Puromycin Aminonucleoside-Induced Nephrosis in Rats. Nephrol. Dial. Transpl..

[97] Yamauchi K., Takano Y., Kasai A., Hayakawa K., Hiramatsu N., Enomoto N., Yao J., Kitamura M. (2006). Screening and Identification of Substances That Regulate Nephrin Gene Expression Using Engineered Reporter Podocytes. Kidney Int..

[98] Liu G., Kaw B., Kurfis J., Rahmanuddin S., Kanwar Y.S., Chugh S.S. (2003). Neph1 and Nephrin Interaction in the Slit Diaphragm Is an Important Determinant of Glomerular Permeability. J. Clin. Investig..

[99] Patrakka J., Tryggvason K. (2007). Nephrin—A Unique Structural and Signaling Protein of the Kidney Filter. Trends Mol. Med..

[100] Garofalo C., Secondulfo C., Apicella L., Bilancio G., De Nicola L., Minutolo R., Borrelli S., Provenzano M., Luciani R., Bellizzi V. (2022). Antiproteinuric Effect of Paricalcitol in Kidney Transplant Recipients with Severe Proteinuria: A Prospective Cohort Study. J. Nephrol..

[101] Alborzi P., Patel N.A., Peterson C., Bills J.E., Bekele D.M., Bunaye Z., Light R.P., Agarwal R. (2008). Paricalcitol Reduces Albuminuria and Inflammation in Chronic Kidney Disease: A Randomized Double-Blind Pilot Trial. Hypertension.

[102] Fishbane S., Chittineni H., Packman M., Dutka P., Ali N., Durie N. (2009). Oral Paricalcitol in the Treatment of Patients With CKD and Proteinuria: A Randomized Trial. Am. J. Kidney Dis..

[103] De Zeeuw D., Agarwal R., Amdahl M., Audhya P., Coyne D., Garimella T., Parving H.-H., Pritchett Y., Remuzzi G., Ritz E. (2010). Selective Vitamin D Receptor Activation with Paricalcitol for Reduction of Albuminuria in Patients with Type 2 Diabetes (VITAL Study): A Randomised Controlled Trial. Lancet.

[104] Agarwal R. (2009). Vitamin D, Proteinuria, Diabetic Nephropathy, and Progression of CKD. Clin. J. Am. Soc. Nephrol..

[105] Tan X., Wen X., Liu Y. (2008). Paricalcitol Inhibits Renal Inflammation by Promoting Vitamin D Receptor–Mediated Sequestration of NF-κB Signaling. J. Am. Soc. Nephrol..

[106] Kheirouri S., Alizadeh M. (2020). Vitamin D and Advanced Glycation End Products and Their Receptors. Pharmacol. Res..

[107] Fukami K., Taguchi K., Yamagishi S., Okuda S. (2015). Receptor for Advanced Glycation Endproducts and Progressive Kidney Disease. Curr. Opin. Nephrol. Hypertens..

[108] Visco V., Izzo C., Bonadies D., Di Feo F., Caliendo G., Loria F., Mancusi C., Chivasso P., Di Pietro P., Virtuoso N. (2023). Interventions to Address Cardiovascular Risk in Obese Patients: Many Hands Make Light Work. J. Cardiovasc. Dev. Dis..

[109] Mathieu C., Adorini L. (2002). The Coming of Age of 1,25-Dihydroxyvitamin D3 Analogs as Immunomodulatory Agents. Trends Mol. Med..

[110] Penna G., Adorini L. (2000). 1α,25-Dihydroxyvitamin D3 Inhibits Differentiation, Maturation, Activation, and Survival of Dendritic Cells Leading to Impaired Alloreactive T Cell Activation. J. Immunol..

[111] Sparaco M., Bonavita S. (2024). Vitamin D Supplementation: Effect on Cytokine Profile in Multiple Sclerosis. JCM.

[112] Yeh W.Z., Lea R., Stankovich J., Sampangi S., Laverick L., Van Der Walt A., Jokubaitis V., Gresle M., Butzkueven H. (2024). Transcriptomics Identifies Blunted Immunomodulatory Effects of Vitamin D in People with Multiple Sclerosis. Sci. Rep..

[113] Wei Y., Wang T., Nie X., Shi Z., Liu Z., Zeng Y., Pan R., Zhang R., Deng Y., Li D. (2023). 1,25-Dihydroxyvitamin D3 Provides Benefits in Vitiligo Based on Modulation of CD8+ T Cell Glycolysis and Function. Nutrients.

[114] Liu W.-C., Zheng C.-M., Lu C.-L., Lin Y.-F., Shyu J.-F., Wu C.-C., Lu K.-C. (2015). Vitamin D and Immune Function in Chronic Kidney Disease. Clin. Chim. Acta.

[115] Széles L., Keresztes G., Töröcsik D., Balajthy Z., Krenács L., Póliska S., Steinmeyer A., Zuegel U., Pruenster M., Rot A. (2009). 1,25-Dihydroxyvitamin D3 Is an Autonomous Regulator of the Transcriptional Changes Leading to a Tolerogenic Dendritic Cell Phenotype. J. Immunol..

[116] Sterling K.A., Eftekhari P., Girndt M., Kimmel P.L., Raj D.S. (2012). The Immunoregulatory Function of Vitamin D: Implications in Chronic Kidney Disease. Nat. Rev. Nephrol..

[117] Korf H., Wenes M., Stijlemans B., Takiishi T., Robert S., Miani M., Eizirik D.L., Gysemans C., Mathieu C. (2012). 1,25-Dihydroxyvitamin D3 Curtails the Inflammatory and T Cell Stimulatory Capacity of Macrophages through an IL-10-Dependent Mechanism. Immunobiology.

[118] Morán-Auth Y., Penna-Martinez M., Shoghi F., Ramos-Lopez E., Badenhoop K. (2013). Vitamin D Status and Gene Transcription in Immune Cells. J. Steroid Biochem. Mol. Biol..

[119] Kim H., Kang S.-W., Yoo T.-H., Kim M.S., Kim S.I., Kim Y.S., Choi K.H. (2012). The Impact of Pretransplant 25-Hydroxy Vitamin D Deficiency on Subsequent Graft Function: An Observational Study. BMC Nephrol..

[120] Bienaimé F., Girard D., Anglicheau D., Canaud G., Souberbielle J.C., Kreis H., Noël L.H., Friedlander G., Elie C., Legendre C. (2013). Vitamin D Status and Outcomes after Renal Transplantation. J. Am. Soc. Nephrol..

[121] Lee J.R., Dadhania D., August P., Lee J.B., Suthanthiran M., Muthukumar T. (2014). Circulating Levels of 25-Hydroxyvitamin D and Acute Cellular Rejection in Kidney Allograft Recipients. Transplantation.

[122] Keyzer C.A., Riphagen I.J., Joosten M.M., Navis G., Muller Kobold A.C., Kema I.P., Bakker S.J.L., de Borst M.H., NIGRAM Consortium (2015). Associations of 25(OH) and 1,25(OH)2 Vitamin D with Long-Term Outcomes in Stable Renal Transplant Recipients. J. Clin. Endocrinol. Metab..

[123] Wood D. (2001). Established and Emerging Cardiovascular Risk Factors. Am. Heart J..

[124] Izzo C., Visco V., Gambardella J., Ferruzzi G.J., Rispoli A., Rusciano M.R., Toni A.L., Virtuoso N., Carrizzo A., Di Pietro P. (2023). Cardiovascular Implications of microRNAs in Coronavirus Disease 2019. J. Pharmacol. Exp. Ther..

[125] Williams B., Mancia G., Spiering W., Agabiti Rosei E., Azizi M., Burnier M., Clement D.L., Coca A., de Simone G., Dominiczak A. (2018). 2018 ESC/ESH Guidelines for the Management of Arterial Hypertension: The Task Force for the Management of Arterial Hypertension of the European Society of Cardiology (ESC) and the European Society of Hypertension (ESH). Eur. Heart J..

[126] Legarth C., Grimm D., Krüger M., Infanger M., Wehland M. (2019). Potential Beneficial Effects of Vitamin D in Coronary Artery Disease. Nutrients.

[127] Rai V., Agrawal D.K. (2017). Role of Vitamin D in Cardiovascular Diseases. Endocrinol. Metab. Clin. N. Am..

[128] Wimalawansa S.J. (2018). Vitamin D and Cardiovascular Diseases: Causality. J. Steroid Biochem. Mol. Biol..

[129] McMullan C.J., Borgi L., Curhan G.C., Fisher N., Forman J.P. (2017). The Effect of Vitamin D on Renin-Angiotensin System Activation and Blood Pressure: A Randomized Control Trial. J. Hypertens..

[130] Carrizzo A., Moltedo O., Damato A., Martinello K., Di Pietro P., Oliveti M., Acernese F., Giugliano G., Izzo R., Sommella E. (2020). New Nutraceutical Combination Reduces Blood Pressure and Improves Exercise Capacity in Hypertensive Patients Via a Nitric Oxide-Dependent Mechanism. J. Am. Heart Assoc..

[131] Lavie C.J., DiNicolantonio J.J., Milani R.V., O’Keefe J.H. (2013). Vitamin D and Cardiovascular Health. Circulation.

[132] Jorde R., Sundsfjord J., Haug E., Bønaa K.H. (2000). Relation Between Low Calcium Intake, Parathyroid Hormone, and Blood Pressure. Hypertension.

[133] Barbarawi M., Kheiri B., Zayed Y., Barbarawi O., Dhillon H., Swaid B., Yelangi A., Sundus S., Bachuwa G., Alkotob M.L. (2019). Vitamin D Supplementation and Cardiovascular Disease Risks in More Than 83 000 Individuals in 21 Randomized Clinical Trials: A Meta-Analysis. JAMA Cardiol..

[134] De Boer I.H., Kestenbaum B., Shoben A.B., Michos E.D., Sarnak M.J., Siscovick D.S. (2009). 25-Hydroxyvitamin D Levels Inversely Associate with Risk for Developing Coronary Artery Calcification. J. Am. Soc. Nephrol..

[135] Zehnder D., Bland R., Chana R.S., Wheeler D.C., Howie A.J., Williams M.C., Stewart P.M., Hewison M. (2002). Synthesis of 1,25-Dihydroxyvitamin D3 by Human Endothelial Cells Is Regulated by Inflammatory Cytokines: A Novel Autocrine Determinant of Vascular Cell Adhesion. J. Am. Soc. Nephrol..

[136] De Falco E., Carnevale R., Pagano F., Chimenti I., Fianchini L., Bordin A., Siciliano C., Monticolo R., Equitani F., Carrizzo A. (2016). Role of NOX2 in Mediating Doxorubicin-Induced Senescence in Human Endothelial Progenitor Cells. Mech. Ageing Dev..

[137] Dao H., Essalihi R., Bouvet C., Moreau P. (2005). Evolution and Modulation of Age-Related Medial Elastocalcinosis: Impact on Large Artery Stiffness and Isolated Systolic Hypertension. Cardiovasc. Res..

[138] Jono S., Nishizawa Y., Shioi A., Morii H. (1998). 1,25-Dihydroxyvitamin D_3_ Increases In Vitro Vascular Calcification by Modulating Secretion of Endogenous Parathyroid Hormone–Related Peptide. Circulation.

[139] Yamamoto T., Kozawa O., Tanabe K., Akamatsu S., Matsuno H., Dohi S., Hirose H., Uematsu T. (2002). 1,25-Dihydroxyvitamin D3 Stimulates Vascular Endothelial Growth Factor Release in Aortic Smooth Muscle Cells: Role of P38 Mitogen-Activated Protein Kinase. Arch. Biochem. Biophys..

[140] Di Pietro P., Abate A.C., Prete V., Damato A., Venturini E., Rusciano M.R., Izzo C., Visco V., Ciccarelli M., Vecchione C. (2024). C2CD4B Evokes Oxidative Stress and Vascular Dysfunction via a PI3K/Akt/PKCα-Signaling Pathway. Antioxidants.

[141] Jensen N.S., Wehland M., Wise P.M., Grimm D. (2023). Latest Knowledge on the Role of Vitamin D in Hypertension. Int. J. Mol. Sci..

[142] Glass C.K., Witztum J.L. (2001). Atherosclerosis. Cell.

[143] Di Pietro P., Izzo C., Abate A.C., Iesu P., Rusciano M.R., Venturini E., Visco V., Sommella E., Ciccarelli M., Carrizzo A. (2023). The Dark Side of Sphingolipids: Searching for Potential Cardiovascular Biomarkers. Biomolecules.

[144] Di Pietro P., Lizio R., Izzo C., Visco V., Damato A., Venturini E., De Lucia M., Galasso G., Migliarino S., Rasile B. (2022). A Novel Combination of High-Load Omega-3 Lysine Complex (AvailOm®) and Anthocyanins Exerts Beneficial Cardiovascular Effects. Antioxidants.

[145] Falk E. (2006). Pathogenesis of Atherosclerosis. J. Am. Coll. Cardiol..

[146] Pál É., Ungvári Z., Benyó Z., Várbíró S. (2023). Role of Vitamin D Deficiency in the Pathogenesis of Cardiovascular and Cerebrovascular Diseases. Nutrients.

[147] Andrukhova O., Slavic S., Zeitz U., Riesen S.C., Heppelmann M.S., Ambrisko T.D., Markovic M., Kuebler W.M., Erben R.G. (2014). Vitamin D Is a Regulator of Endothelial Nitric Oxide Synthase and Arterial Stiffness in Mice. Mol. Endocrinol..

[148] Carthy E.P., Yamashita W., Hsu A., Ooi B.S. (1989). 1,25-Dihydroxyvitamin D3 and Rat Vascular Smooth Muscle Cell Growth. Hypertension.

[149] Raymond M.-A., Désormeaux A., Labelle A., Soulez M., Soulez G., Langelier Y., Pshezhetsky A.V., Hébert M.-J. (2005). Endothelial Stress Induces the Release of Vitamin D-Binding Protein, a Novel Growth Factor. Biochem. Biophys. Res. Commun..

[150] Bobryshev Y.V. (2010). Vitamin D_3_ Suppresses Immune Reactions in Atherosclerosis, Affecting Regulatory T Cells and Dendritic Cell Function. ATVB.

[151] Watson K.E., Abrolat M.L., Malone L.L., Hoeg J.M., Doherty T., Detrano R., Demer L.L. (1997). Active Serum Vitamin D Levels Are Inversely Correlated With Coronary Calcification. Circulation.

[152] Roger V.L. (2021). Epidemiology of Heart Failure. Circ. Res..

[153] Schwinger R.H.G. (2021). Pathophysiology of Heart Failure. Cardiovasc. Diagn. Ther..

[154] Hazique M., Khan K.I., Ramesh P., Kanagalingam S., Zargham Ul Haq F., Victory Srinivasan N., Khan A.I., Mashat G.D., Khan S. (2022). A Study of Vitamin D and Its Correlation With Severity and Complication of Congestive Heart Failure: A Systematic Review. Cureus.

[155] Campanile A., Visco V., De Carlo S., Ferruzzi G.J., Mancusi C., Izzo C., Mongiello F., Di Pietro P., Virtuoso N., Ravera A. (2023). Sacubitril/Valsartan vs. Standard Medical Therapy on Exercise Capacity in HFrEF Patients. Life.

[156] Qu H., Lin K., Wang H., Wei H., Ji B., Yang Z., Peng C., Xiao X., Deng H. (2017). 1,25(OH)2D3 Improves Cardiac Dysfunction, Hypertrophy, and Fibrosis through PARP1/SIRT1/mTOR-Related Mechanisms in Type 1 Diabetes. Mol. Nutr. Food Res..

[157] Chen S., Law C.S., Grigsby C.L., Olsen K., Hong T.-T., Zhang Y., Yeghiazarians Y., Gardner D.G. (2011). Cardiomyocyte-Specific Deletion of the Vitamin D Receptor Gene Results in Cardiac Hypertrophy. Circulation.

[158] Schroten N.F., Ruifrok W.P.T., Kleijn L., Dokter M.M., Silljé H.H., Lambers Heerspink H.J., Bakker S.J.L., Kema I.P., Van Gilst W.H., Van Veldhuisen D.J. (2013). Short-Term Vitamin D3 Supplementation Lowers Plasma Renin Activity in Patients with Stable Chronic Heart Failure: An Open-Label, Blinded End Point, Randomized Prospective Trial (VitD-CHF Trial). Am. Heart J..

[159] Mahmood S.S., Levy D., Vasan R.S., Wang T.J. (2014). The Framingham Heart Study and the Epidemiology of Cardiovascular Disease: A Historical Perspective. Lancet.

[160] Brøndum-Jacobsen P., Benn M., Jensen G.B., Nordestgaard B.G. (2012). 25-Hydroxyvitamin d Levels and Risk of Ischemic Heart Disease, Myocardial Infarction, and Early Death: Population-Based Study and Meta-Analyses of 18 and 17 Studies. Arter. Thromb. Vasc. Biol..

[161] Ameri P., Canepa M., Milaneschi Y., Spallarossa P., Leoncini G., Giallauria F., Strait J.B., Lakatta E.G., Brunelli C., Murialdo G. (2013). Relationship between Vitamin D Status and Left Ventricular Geometry in a Healthy Population: Results from the Baltimore Longitudinal Study of Aging. J. Intern. Med..

[162] Şeker T., Gür M., Uçar H., Türkoğlu C., Oytun Baykan A., Özaltun B., Harbalıoğlu H., Yüksel Kalkan G., Kaypaklı O., Kuloğlu O. (2015). Lower Serum 25-Hydroxyvitamin D Level Is Associated with Impaired Myocardial Performance and Left Ventricle Hypertrophy in Newly Diagnosed Hypertensive Patients. Anatol. J. Cardiol..

[163] Moretti H.D., Colucci V.J., Berry B.D. (2017). Vitamin D3 Repletion versus Placebo as Adjunctive Treatment of Heart Failure Patient Quality of Life and Hormonal Indices: A Randomized, Double-Blind, Placebo-Controlled Trial. BMC Cardiovasc. Disord..

[164] Hahn J., Cook N.R., Alexander E.K., Friedman S., Walter J., Bubes V., Kotler G., Lee I.-M., Manson J.E., Costenbader K.H. (2022). Vitamin D and Marine Omega 3 Fatty Acid Supplementation and Incident Autoimmune Disease: VITAL Randomized Controlled Trial. BMJ.

[165] Scragg R., Stewart A.W., Waayer D., Lawes C.M.M., Toop L., Sluyter J., Murphy J., Khaw K.-T., Camargo C.A. (2017). Effect of Monthly High-Dose Vitamin D Supplementation on Cardiovascular Disease in the Vitamin D Assessment Study: A Randomized Clinical Trial. JAMA Cardiol..

[166] Zittermann A., Ernst J.B., Prokop S., Fuchs U., Dreier J., Kuhn J., Knabbe C., Birschmann I., Schulz U., Berthold H.K. (2017). Effect of Vitamin D on All-Cause Mortality in Heart Failure (EVITA): A 3-Year Randomized Clinical Trial with 4000 IU Vitamin D Daily. Eur. Heart J..

[167] Avenell A., MacLennan G.S., Jenkinson D.J., McPherson G.C., McDonald A.M., Pant P.R., Grant A.M., Campbell M.K., Anderson F.H., Cooper C. (2012). Long-Term Follow-Up for Mortality and Cancer in a Randomized Placebo-Controlled Trial of Vitamin D3 and/or Calcium (RECORD Trial). J. Clin. Endocrinol. Metab..

[168] Witte K.K., Byrom R., Gierula J., Paton M.F., Jamil H.A., Lowry J.E., Gillott R.G., Barnes S.A., Chumun H., Kearney L.C. (2016). Effects of Vitamin D on Cardiac Function in Patients With Chronic HF: The VINDICATE Study. J. Am. Coll. Cardiol..

[169] Gómez-Outes A., Lagunar-Ruíz J., Terleira-Fernández A.-I., Calvo-Rojas G., Suárez-Gea M.L., Vargas-Castrillón E. (2016). Causes of Death in Anticoagulated Patients with Atrial Fibrillation. J. Am. Coll. Cardiol..

[170] Andrade J., Khairy P., Dobrev D., Nattel S. (2014). The Clinical Profile and Pathophysiology of Atrial Fibrillation: Relationships among Clinical Features, Epidemiology, and Mechanisms. Circ. Res..

[171] Visco V., Izzo C., Mancusi C., Rispoli A., Tedeschi M., Virtuoso N., Giano A., Gioia R., Melfi A., Serio B. (2023). Artificial Intelligence in Hypertension Management: An Ace up Your Sleeve. J. Cardiovasc. Dev. Dis..

[172] Qayyum F., Landex N.L., Agner B.R., Rasmussen M., Jøns C., Dixen U. (2012). Vitamin D Deficiency Is Unrelated to Type of Atrial Fibrillation and Its Complications. Dan. Med. J..

[173] Smith M.B., May H.T., Blair T.L., Anderson J.L., Muhlestein J.B., Horne B.D., Lappe D.L., Day J.D., Crandall B.G., Weiss P. (2011). Abstract 14699: Vitamin D Excess Is Significantly Associated with Risk of Atrial Fibrillation. Circulation.

[174] Thompson J., Nitiahpapand R., Bhatti P., Kourliouros A. (2015). Vitamin D Deficiency and Atrial Fibrillation. Int. J. Cardiol..

[175] Liu S., Tang W., Zhou J., Stubbs J.R., Luo Q., Pi M., Quarles L.D. (2006). Fibroblast Growth Factor 23 Is a Counter-Regulatory Phosphaturic Hormone for Vitamin D. J. Am. Soc. Nephrol..

[176] Nakamura K., Isoyama N., Nakayama Y., Hiroyoshi T., Fujikawa K., Miura Y., Kurosu H., Matsuyama H., Kuro-o M. (2022). Association between Amorphous Calcium-Phosphate Ratios in Circulating Calciprotein Particles and Prognostic Biomarkers in Hemodialysis Patients. Sci. Rep..

[177] Faul C., Amaral A.P., Oskouei B., Hu M.-C., Sloan A., Isakova T., Gutiérrez O.M., Aguillon-Prada R., Lincoln J., Hare J.M. (2011). FGF23 Induces Left Ventricular Hypertrophy. J. Clin. Investig..

[178] Havakuk O., Entin-Meer M., Ben-Shoshan J., Goryainov P., Maysel-Auslender S., Joffe E., Keren G. (2013). Effect of Vitamin D Analogues on Acute Cardiorenal Syndrome: A Laboratory Rat Model. Isr. Med. Assoc. J..

[179] Darabian S., Rattanasompattikul M., Hatamizadeh P., Bunnapradist S., Budoff M.J., Kovesdy C.P., Kalantar-Zadeh K. (2012). Cardiorenal Syndrome and Vitamin D Receptor Activation in Chronic Kidney Disease. Kidney Res. Clin. Pract..

[180] Bodyak N., Ayus J.C., Achinger S., Shivalingappa V., Ke Q., Chen Y.-S., Rigor D.L., Stillman I., Tamez H., Kroeger P.E. (2007). Activated Vitamin D Attenuates Left Ventricular Abnormalities Induced by Dietary Sodium in Dahl Salt-Sensitive Animals. Proc. Natl. Acad. Sci. USA.

[181] Wu-Wong J.R., Noonan W., Nakane M., Brooks K.A., Segreti J.A., Polakowski J.S., Cox B. (2010). Vitamin d Receptor Activation Mitigates the Impact of Uremia on Endothelial Function in the 5/6 Nephrectomized Rats. Int. J. Endocrinol..

[182] Teng M., Wolf M., Lowrie E., Ofsthun N., Lazarus J.M., Thadhani R. (2003). Survival of Patients Undergoing Hemodialysis with Paricalcitol or Calcitriol Therapy. N. Engl. J. Med..

[183] Tentori F., Hunt W.C., Stidley C.A., Rohrscheib M.R., Bedrick E.J., Meyer K.B., Johnson H.K., Zager P.G., Medical Directors of Dialysis Clinic Inc (2006). Mortality Risk among Hemodialysis Patients Receiving Different Vitamin D Analogs. Kidney Int..

[184] Manson J.E., Bassuk S.S., Buring J.E., VITAL Research Group (2020). Principal Results of the VITamin D and OmegA-3 TriaL (VITAL) and Updated Meta-Analyses of Relevant Vitamin D Trials. J. Steroid Biochem. Mol. Biol..

[185] Nehgme R., Fahey J.T., Smith C., Carpenter T.O. (1997). Cardiovascular Abnormalities in Patients with X-Linked Hypophosphatemia. J. Clin. Endocrinol. Metab..

[186] Sattar N., Welsh P., Panarelli M., Forouhi N.G. (2012). Increasing Requests for Vitamin D Measurement: Costly, Confusing, and without Credibility. Lancet.

[187] Martineau A.R., Jolliffe D.A., Hooper R.L., Greenberg L., Aloia J.F., Bergman P., Dubnov-Raz G., Esposito S., Ganmaa D., Ginde A.A. (2017). Vitamin D Supplementation to Prevent Acute Respiratory Tract Infections: Systematic Review and Meta-Analysis of Individual Participant Data. BMJ.

[188] Domazet Bugarin J., Dosenovic S., Ilic D., Delic N., Saric I., Ugrina I., Stojanovic Stipic S., Duplancic B., Saric L. (2023). Vitamin D Supplementation and Clinical Outcomes in Severe COVID-19 Patients-Randomized Controlled Trial. Nutrients.

[189] Subramanian S., Griffin G., Hewison M., Hopkin J., Kenny R.A., Laird E., Quinton R., Thickett D., Rhodes J.M. (2022). Vitamin D and COVID-19-Revisited. J. Intern. Med..

[190] Junarta J., Jha V., Banerjee D. (2019). Insight into the Impact of Vitamin D on Cardiovascular Outcomes in Chronic Kidney Disease. Nephrology.

[191] Cheah S., English D.R., Harrison S.J., Vajdic C.M., Giles G.G., Milne R.L. (2023). Sunlight, Vitamin D, Vitamin D Receptor Polymorphisms, and Risk of Multiple Myeloma: A Systematic Review. Cancer Epidemiol..

[192] Jagannath V.A., Filippini G., Di Pietrantonj C., Asokan G.V., Robak E.W., Whamond L., Robinson S.A. (2018). Vitamin D for the Management of Multiple Sclerosis. Cochrane Database Syst. Rev..

[193] Harrison S.R., Li D., Jeffery L.E., Raza K., Hewison M. (2020). Vitamin D, Autoimmune Disease and Rheumatoid Arthritis. Calcif Tissue Int..

[194] Gorter E.A., Hamdy N.A.T., Appelman-Dijkstra N.M., Schipper I.B. (2014). The Role of Vitamin D in Human Fracture Healing: A Systematic Review of the Literature. Bone.

[195] Sprague S., Bhandari M., Devji T., Scott T., Petrisor B., McKay P., Slobogean G.P. (2016). Prescription of Vitamin D to Fracture Patients: A Lack of Consensus and Evidence. J. Orthop. Trauma.

[196] Rosenberg K. (2022). Supplemental Vitamin D Doesn’t Reduce Risk of Fracture in Healthy Older Adults. Am. J. Nurs..

[197] Reid I.R., Bolland M.J., Grey A. (2014). Effects of Vitamin D Supplements on Bone Mineral Density: A Systematic Review and Meta-Analysis. Lancet.

[198] Mann M.C., Exner D.V., Hemmelgarn B.R., Hanley D.A., Turin T.C., MacRae J.M., Wheeler D.C., Sola D.Y., Ramesh S., Ahmed S.B. (2016). The VITAH Trial-Vitamin D Supplementation and Cardiac Autonomic Tone in Patients with End-Stage Kidney Disease on Hemodialysis: A Blinded, Randomized Controlled Trial. Nutrients.

[199] Lappe J., Watson P., Travers-Gustafson D., Recker R., Garland C., Gorham E., Baggerly K., McDonnell S.L. (2017). Effect of Vitamin D and Calcium Supplementation on Cancer Incidence in Older Women: A Randomized Clinical Trial. JAMA.

[200] Atkinson M.A., Juraschek S.P., Bertenthal M.S., Detrick B., Furth S.L., Miller E.R. (2017). Pilot Study of the Effect of Cholecalciferol Supplementation on Hepcidin in Children with Chronic Kidney Disease: Results of the D-Fense Trial. Pediatr. Nephrol.

[201] Theodoratou E., Tzoulaki I., Zgaga L., Ioannidis J.P.A. (2014). Vitamin D and Multiple Health Outcomes: Umbrella Review of Systematic Reviews and Meta-Analyses of Observational Studies and Randomised Trials. BMJ.

[202] Thompson B., Waterhouse M., English D.R., McLeod D.S., Armstrong B.K., Baxter C., Duarte Romero B., Ebeling P.R., Hartel G., Kimlin M.G. (2023). Vitamin D Supplementation and Major Cardiovascular Events: D-Health Randomised Controlled Trial. BMJ.

[203] Greaves J.H., Redfern R., King R.E. (1974). Some Properties of Calciferol as a Rodenticide. J. Hyg..

[204] Davies J.S., Poole C.D., Feldschreiber P. (2014). The Medico-Legal Aspects of Prescribing Vitamin D. Br. J. Clin. Pharmacol..

[205] Vieth R. (2007). Vitamin D Toxicity, Policy, and Science. J. Bone Miner. Res..

[206] Lim K., Thadhani R. (2020). Vitamin D Toxicity. J. Bras. Nefrol..

[207] Taylor P.N., Davies J.S. (2018). A Review of the Growing Risk of Vitamin D Toxicity from Inappropriate Practice. Br. J. Clin. Pharmacol..

[208] Cirillo M., Bilancio G., Cirillo C. (2016). Reversible Vascular Calcifications Associated with Hypervitaminosis D. J. Nephrol..

[209] Chiricone D., De Santo N.G., Cirillo M. (2003). Unusual Cases of Chronic Intoxication by Vitamin D. J. Nephrol..

[210] Bargagli M., Ferraro P.M., Vittori M., Lombardi G., Gambaro G., Somani B. (2021). Calcium and Vitamin D Supplementation and Their Association with Kidney Stone Disease: A Narrative Review. Nutrients.

[211] Ferraro P.M., Bargagli M., Trinchieri A., Gambaro G. (2020). Risk of Kidney Stones: Influence of Dietary Factors, Dietary Patterns, and Vegetarian–Vegan Diets. Nutrients.

[212] Casmir F. (1975). The Journal of State Medicine. Volume XX: 341–368, 1912. The Etiology of the Deficiency Diseases, Beri-Beri, Polyneuritis in Birds, Epidemic Dropsy, Scurvy, Experimental Scurvy in Animals, Infantile Scurvy, Ship Beri-Beri, Pellagra. Nutr. Rev..

[213] Santulli G., Pascale V., Finelli R., Visco V., Giannotti R., Massari A., Morisco C., Ciccarelli M., Illario M., Iaccarino G. (2019). We Are What We Eat: Impact of Food from Short Supply Chain on Metabolic Syndrome. J. Clin. Med..

[214] Marcén R., Jimenez S., Fernández-Rodriguez A., Galeano C., Villafruela J.J., Gomis A., Teruel J.L., Quereda C. (2012). Are Low Levels of 25-Hydroxyvitamin D a Risk Factor for Cardiovascular Diseases or Malignancies in Renal Transplantation?. Nephrol. Dial. Transpl..

[215] Muscogiuri G., Altieri B., Annweiler C., Balercia G., Pal H.B., Boucher B.J., Cannell J.J., Foresta C., Grübler M.R., Kotsa K. (2017). Vitamin D and Chronic Diseases: The Current State of the Art. Arch. Toxicol..

[216] Marcinowska-Suchowierska E., Kupisz-Urbańska M., Łukaszkiewicz J., Płudowski P., Jones G. (2018). Vitamin D Toxicity—A Clinical Perspective. Front. Endocrinol..

[217] Gunta S.S., Thadhani R.I., Mak R.H. (2013). The Effect of Vitamin D Status on Risk Factors for Cardiovascular Disease. Nat. Rev. Nephrol..

